# Relationship between the thrombospondin-1/Toll-like receptor 4 (TSP1/TLR4) pathway and vitamin D levels in obese and normal weight subjects with different metabolic phenotypes

**DOI:** 10.1186/s12576-023-00887-z

**Published:** 2023-11-14

**Authors:** Eman Y. Khairy, Azza Saad

**Affiliations:** Department of Physiology, Medical Research Institute, Alexandria University, 165, Horreya Avenue, Hadara, Alexandria, Egypt

**Keywords:** Vitamin D, Thrombospondin-1, Toll-like receptor 4, Metabolic phenotypes, Inflammation, Metabolic dysfunction

## Abstract

Thrombospondin-1 (TSP1) contributes to obesity-associated inflammation via activating Toll-like receptor 4 (TLR4). The regulatory role of vitamin D on this pathway has been suggested. This study aimed to investigate the relationship between TSP1/TLR4 pathway and vitamin D in obese and normal weight subjects with different metabolic phenotypes. Thirty obese and thirty normal weight men were selected. Anthropometric parameters and serum TSP1, TLR4, TNF-α, vitamin D, and metabolic profile were determined. Metabolic phenotypes of obese and normal weight subjects were determined. Findings revealed enhanced TSP1/TLR4/TNF-α levels and reduced 25(OH)D levels in obese compared to normal weight subjects and metabolically unhealthy compared to metabolically healthy subjects. TSP1 correlated positively with parameters of unhealthy metabolic profile. TSP1, TLR4 and TNF-α levels significantly negatively correlated with vitamin D levels. In conclusion, vitamin D might exert a regulatory role on TSP1/TLR4 pathway, providing a potential mechanism that links hypovitaminosis D with risk of metabolic dysfunction.

## Introduction

Obesity is a serious public health challenge as it contributes to the global burden of several chronic diseases and cardiometabolic complications [1]. Accumulating evidences identify adipose tissue inflammation as a possible mechanism linking obesity with metabolic disorders [2, 3]. Adipose tissue dysfunction causes a pro-inflammatory state as a consequence of an imbalance in adipocytokines production, which is supposed to accelerate cardiometabolic diseases in obese subjects [4].

The concept of heterogeneity between obese subjects regarding their risk for the development of metabolic dysfunctions has been recognized. Several attempts have been developed to categorize individuals based on the degree of obesity and metabolic health resulting in diverse obese and non-obese phenotypes [5]. A subgroup of obese subjects has been found to be more resistant to the inflammatory and metabolic dysfunctions, this is known as metabolic healthy obese (MHO) [6]. Conversely, a subgroup of normal weight subjects may exhibit inflammatory and metabolic abnormalities commonly detected in obese subjects, this is known as the metabolic unhealthy normal weight (MUNW) [5]. Notably, the metabolically unhealthy obese (MUO) persons are characterized by higher degree of inflammation in the visceral adipose tissue as compared to the MHO persons [7].

Thrombospondin-1 (TSP1) is an adipose-derived multifunctional glycoprotein released by several types of cells, including platelets, macrophages and adipocytes [8, 9]. It has been emphasized as a potential mediator of the obesity associated-adipose tissue inflammation and insulin resistance [10]. TSP1 expression was found to be elevated in the adipose tissue of obese and insulin-resistant individuals [10] and in obese mice [11]. TSP1 deficiency in a high fat diet-fed mice was reported to reduce macrophage accumulation in the adipose tissue and to protect against inflammation and insulin resistance induced by obesity [11, 12]. Moreover, the macrophages isolated from TSP1-deficient mice had reduced inflammatory response and chemotactic activity, thus supporting a role for TSP1 in regulating the macrophages function [11].

A suggested mechanism by which TSP1 regulates the macrophage activity and the production of inflammatory cytokines is via activating the Toll-like receptor 4 (TLR4) signaling pathway [13], which is considered as a main trigger of the obesity-induced inflammatory response [14]. TSP1 treatment of the macrophages was found to induce their expression of tumor-necrosis factor (TNF)-α in a dosage and time-dependent manner through activation of the TLR4 signaling pathway [13]. Consequently, it is plausible that TSP1/TLR4-dependent chronic activation of the macrophages in obese subjects may influence the development of metabolic dysregulation.

Obesity is a known risk factor for vitamin D deficiency [15]. Vitamin D, a steroid hormone with calcemic and non-calcemic functions, has been proposed to contribute to the protective metabolic profile found in MHO individuals [16]. Several genomic and non-genomic actions of vitamin D are engaged in the maintenance of insulin sensitivity, among which the immunomodulatory and anti-inflammatory actions [17, 18].

Interestingly, vitamin D has been suggested to have a regulatory effect on TSP1 [19]. Vitamin D supplementation was found to lower TSP1 levels in vitamin D insufficient healthy adults [20], and to downregulate TSP1 mRNA expression and protein secretion in breast cancer cell lines [19]. Besides, an inverse relationship was reported between levels of vitamin D and TSP1 in children with sickle cell anemia [21]. However, data are limited concerning the relation between vitamin D status and TSP1 levels in obesity as well as in the metabolically healthy and unhealthy status.

Therefore, the present study aimed at examining the relationship between the TSP1/TLR4 inflammatory pathway and serum vitamin D levels among obese and normal weight subjects with different metabolic phenotypes, as a possible mechanism by which hypovitaminosis D may impact metabolic health.

## Materials and methods

### Study participants

This study included thirty apparently healthy obese men with body mass index (BMI) ≥ 30 kg/m^2^ and thirty age matched apparently healthy normal weight men (18.5 ≤ BMI ≤ 24.9 kg/m^2^).

History taking and clinical examination were performed to all the participants. All were non-smokers, free from acute infections, malignancies or any viral, parasitic, autoimmune or endocrine diseases and were similarly exposed to the factors that may influence vitamin D levels such as sun exposure and dietary intake. All participants were not receiving any medications nor vitamin supplementation. Written informed consents were provided by all the participants. This study was approved by the Ethics Committee of the Medical Research Institute, Alexandria University, Egypt (IORG0008812).

### Anthropometric measurements

Based on the standards for anthropometric measurements, the weight (in kilograms) was measured in socks and light clothes. Measurement of height was done using a stadiometer in standing position. BMI calculation was conducted as weight divided by height in meters squared (kg/m^2^) [22].

The measurement of waist circumference (WC) was performed using a flexible non-elastic measuring tape applied directly to the skin, midway between the lower costal margin and the iliac crest, at the end of tidal expiration while the subject standing [23].

### Biochemical analysis

Blood samples were obtained from each participant by venipuncture after 12 h of fasting. Samples were left 30 min at room temperature for spontaneous clotting, then centrifuged for 5 min at 3000 rpm. The serum was stored at − 20 °C until biochemical analysis. Fasting glucose concentrations were determined using a glucose hexokinase assay. Serum lipids [triglycerides (TG), high-density lipoprotein cholesterol (HDL-C) and total cholesterol (TC)] were evaluated using enzymatic colorimetric method (BioSystems, Barcelona, Spain). The Friedewald equation was used to calculate the levels of low-density lipoprotein cholesterol (LDL-C) as follows: LDL-C (mg/dL) = TC (mg/dL) − HDL-C (mg/dL) − TG (mg/dL)/5 [24]. High sensitivity C-reactive protein (hs-CRP) was assessed using turbidimetric assay (Linear Chemicals, Montgat, Barcelona, Spain). Fasting insulin was quantified by enzyme-linked immunosorbent assay (ELISA) using a commercial kit (Millipore Corporation, Billerica, USA).

According to Matthews et al. [25], the homeostasis model assessment of insulin resistance (HOMA-IR) index was calculated as follows: fasting insulin (µIU/mL) × fasting blood glucose (mmol/L)/22.5 or fasting insulin (µIU/mL) × fasting glucose (mg/dL)/405.

### Determination of the metabolic health status

The metabolic unhealthy state was defined based on the Karelis criteria [26] of having two or more of the following metabolic risk factors: TG ≥ 1.7 mmol/L or use of lipid-lowering drugs, LDL-C ≥ 2.6 mmol/L, HDL-C ≤ 1.0/1.3 mmol/L for men/women, HOMA-IR ≥ 2.7, or hs-CRP ≥ 3.0 mg/L. Subjects with less than two risk factors were considered as metabolically healthy. Accordingly, study subjects (normal weight and obese) were categorized into four groups:

- Metabolically healthy obese (MHO) group (n = 13).
- Metabolically unhealthy obese (MUO) group (n = 17).
- Metabolically healthy normal weight (MHNW) group (n = 16).
- Metabolically unhealthy normal weight (MUNW) group (n = 14).

### Determination of serum 25-hydroxyvitamin D (25(OH)D) levels

Serum 25(OH)D levels were measured using an ELISA kit (ab213966, Abcam, Cambridge, UK), following manufacturer^’^s instructions. As stated in the Endocrine Society clinical practice guidelines [27], serum 25(OH)D levels are categorized into deficient (< 20 ng/mL), insufficient (20–29.9 ng/mL) or sufficient (30–100 ng/mL).

### Determination of serum thrombospondin-1 (TSP1), Toll-like receptor 4 (TLR-4) and tumor necrosis factor (TNF-α) levels

ELISA kits were used for the quantitative determination of serum TSP1, TLR-4 and TNF-α [TSP1 (R&D Systems, Minneapolis, MN, USA, Catalog # DTSP10), TLR-4 (MyBioSource, San Diego, USA, Catalog # MBS765181) and TNF-α (MyBioSource, San Diego, USA, Catalog # MBS2502004)], following manufacturers^’^ instructions.

### Statistical analysis

Statistical analysis was conducted using Statistical Package for the Social Sciences (SPSS) version 20.0 (Armonk, NY: IBM Corp**)**. The normality of distribution was tested using the Kolmogorov–Smirnov test. Quantitative data were described using mean ± standard error of mean (SEM). Student’s t-test was used to compare the means of normally distributed quantitative variables between two groups, whereas for comparing the four studied groups one way analysis of variance (ANOVA) was used then followed by a Post Hoc test (Tukey) for pairwise comparisons. Pearson’s correlation coefficient test was used to examine the correlations between two quantitative variables. Statistical significance was set at *p* < 0.05.

## Results

As shown in Table 1, the comparison between the normal weight and obese subjects showed significant differences in all the studied parameters (p < 0.001), except for fasting blood glucose.

**Table 1:** Anthropometric and biochemical characteristics of the normal weight and obese subjects

	Normal weight (n = 30)	Obese (n = 30)	t	p
BMI (kg/m^2^)	23.82 ± 0.16	32.60 ± 0.50	16.752	< 0.001*
Waist circumference (cm)	98.03 ± 0.94	117.67 ± 1.80	9.668*	< 0.001*
Total cholesterol (mmol/L)	4.84 ± 0.08	5.58 ± 0.12	5.007*	< 0.001*
Triglyceride (mmol/L)	1.62 ± 0.04	1.92 ± 0.05	4.475*	< 0.001*
HDL-C (mmol/L)	1.02 ± 0.02	0.97 ± 0.01	2.717*	0.009^*^
LDL-C (mmol/L)	3.07 ± 0.08	3.73 ± 0.10	4.978*	< 0.001*
FBG (mg/dL)	88.80 ± 1.94	90.67 ± 1.32	0.796	0.430
Insulin (µIU/mL)	6.82 ± 0.54	13.68 ± 1.41	4.551*	< 0.001*
HOMA-IR	1.53 ± 0.14	3.11 ± 0.34	4.287*	< 0.001*
hs-CRP (mg/L)	1.89 ± 0.18	3.89 ± 0.47	3.969*	< 0.001*
25 (OH)D (ng/mL)	24.89 ± 1.15	10.67 ± 0.59	11.018*	< 0.001*
TSP1 (ng/mL)	10,394.03 ± 466.87	23,586.33 ± 494.15	19.406*	< 0.001*
TLR-4 (ng/mL)	0.71 ± 0.01	1.48 ± 0.06	11.966*	< 0.001*
TNF-α (pg/mL)	8.19 ± 0.15	12.06 ± 0.21	14.825*	< 0.001*

Data was expressed by using mean ± standard error of mean (SEM)

*p* p value for Student’s t-test for comparing between normal weight and obese subjects, *t* t value for Student’s t-test, *BMI* body mass index, *HDL-C* high density lipoprotein cholesterol, *LDL-C* low density lipoprotein cholesterol, *FBG* fasting blood glucose, *HOMA-IR* homeostasis model assessment for insulin resistance, *hs-CRP* high sensitivity C reactive protein, *25(OH)D* 25 hydroxyvitamin D, *TSP1* thrombospondin-1, *TLR-4* Toll like receptor-4, *TNF-α* tumor necrosis factor-α

*Statistical significance at p < 0.05

### Anthropometric and metabolic health profile results

According to the metabolic health, the comparison of the normal weight and obese healthy and unhealthy subgroups was represented in Table 2. Using ANOVA test, significant differences were noticed between groups in all the anthropometric and the metabolic profile measurements (p < 0.001), except for the fasting blood glucose. Significantly higher levels of serum triglyceride, LDL-C and total cholesterol as well as significantly lower serum level of HDL-C were observed in the metabolically unhealthy compared with the metabolically healthy obese and normal weight subjects (p < 0.001). HOMA index and the serum insulin and hs-CRP levels were elevated in the metabolically unhealthy subjects in comparison to their metabolically healthy counterparts; however, this reached the level of significance only among the obese subjects (p < 0.001).

**Table 2:** Anthropometric characteristics and metabolic profile in the normal weight and obese subjects stratified by metabolic health

	Normal weight (n = 30)		Obese subject (n = 30)		F	p
	MHNW (n = 16)	MUNW (n = 14)	MHO (n = 13)	MUO (n = 17)		
BMI (kg/m^2^)	23.42 ± 0.25	24.28 ± 0.12	31.95^ab^ ± 0.51	33.09^ab^ ± 0.78	97.656*	< 0.001*
Waist circumference (cm)	95.56 ± 0.87	100.86 ± 1.45	113.62^ab^ ± 2.34	120.76^abc^ ± 2.41	39.599*	< 0.001*
Total cholesterol (mmol/L)	4.52 ± 0.05	5.22^a^ ± 0.08	5.04^a^ ± 0.04	5.98^abc^ ± 0.15	39.838*	< 0.001*
Triglyceride (mmol/L)	1.48 ± 0.03	1.79^a^ ± 0.03	1.67^a^ ± 0.04	2.10^abc^ ± 0.06	38.726*	< 0.001*
HDL-C (mmol/L)	1.10 ± 0.02	0.94^a^ ± 0.01	1.02^ab^ ± 0.01	0.93^ac^ ± 0.01	52.264*	< 0.001*
LDL-C (mmol/L)	2.74 ± 0.05	3.46^a^ ± 0.08	3.26^a^ ± 0.03	4.09^abc^ ± 0.13	46.470*	< 0.001*
FBG (mg/dL)	84.88 ± 2.62	93.29 ± 2.46	90.77 ± 2.18	90.59 ± 1.67	2.549	0.065
Insulin (µIU/mL)	5.28 ± 0.57	8.58 ± 0.71	8.63 ± 0.64	17.54^abc^ ± 1.99	19.575*	< 0.001*
HOMA-IR	1.11 ± 0.13	2.02 ± 0.20	1.96 ± 0.16	3.99^abc^ ± 0.49	17.014*	< 0.001*
hs-CRP (mg/L)	1.59 ± 0.18	2.23 ± 0.29	2.03 ± 0.22	5.32^abc^ ± 0.63	19.122*	< 0.001*

Data was expressed as mean ± standard error of mean (SEM). Post Hoc Test (Tukey) was used for pairwise comparison between each 2 groups

*MHNW* metabolically healthy normal weight, *MUNW* metabolically unhealthy normal weight, *MHO* metabolically healthy obese, *MUO* metabolically unhealthy obese, *F* F for ANOVA test, *p* p value for comparing all the studied groups, *BMI* Body mass index,* FBG* fasting blood glucose, *HDL-C* high density lipoprotein cholesterol, *hs-CRP* high sensitivity C reactive protein, *HOMA-IR* homeostasis model assessment for insulin resistance, *LDL-C* low density lipoprotein cholesterol

*Statistical significance at p < 0.05

^a^p < 0.05 vs. MHNW

^b^p < 0.05 vs. MUNW

^c^p < 0.05 vs. MHO

### Serum 25(OH)D, TSP1, TLR-4 and TNF-α results

Using ANOVA test, significant differences in the mean values of serum 25(OH)D, TSP1, TLR-4 and TNF-α were noticed between the 4 studied groups (p < 0.001). F values were 133.472, 438.506, 259.100 and 404.342 for serum 25(OH)D, TSP1, TLR-4 and TNF-α, respectively.

**Fig. 1:**
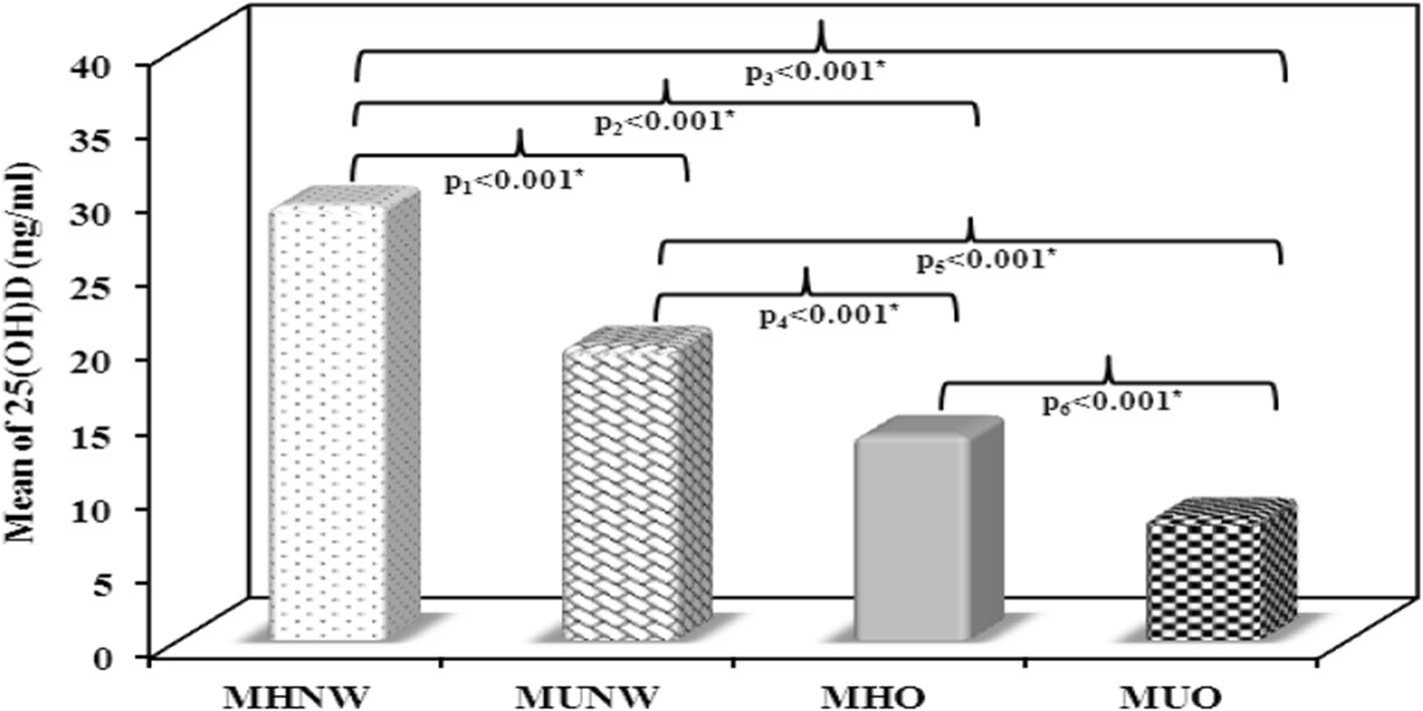
Comparison between the mean values of serum 25(OH)D (ng/mL) in the studied groups. MHNW Metabolically healthy normal weight. MUNW Metabolically unhealthy normal weight. MHO Metabolically healthy obese. MUO Metabolically unhealthy obese. ANOVA test, post hoc test (Tukey) was used for pairwise comparison. p_1_ significant difference MUNW vs. MHNW. p_2_ significant difference MHO vs. MHNW. p_3_ significant difference MUO vs. MHNW. p_4_ significant difference MHO vs. MUNW. p_5_ significant difference MUO vs. MUNW. p_6_ significant difference MUO vs. MHO. *Statistical significance at p < 0.05

**Fig. 2:**
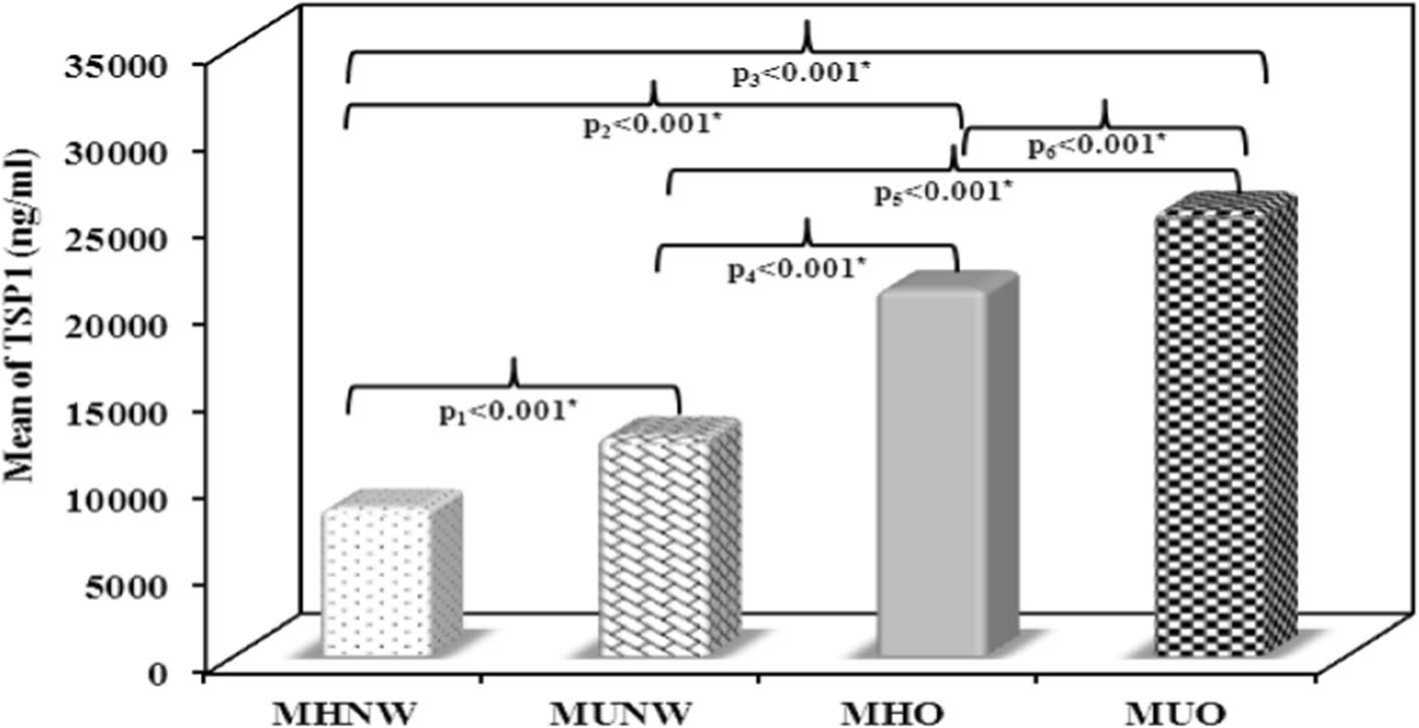
Comparison between the mean values of serum thrombospondin-1 (TSP1) (ng/mL) in the studied groups. MHNW Metabolically healthy normal weight. MUNW Metabolically unhealthy normal weight. MHO Metabolically healthy obese. MUO Metabolically unhealthy obese. ANOVA test, post hoc test (Tukey) was used for pairwise comparison. p_1_ significant difference MUNW vs. MHNW. p_2_ significant difference MHO vs. MHNW. p_3_ significant difference MUO vs. MHNW. p_4_ significant difference MHO vs. MUNW. p_5_ significant difference MUO vs. MUNW. p_6_ significant difference MUO vs. MHO. *Statistical significance at p < 0.05

**Fig. 3:**
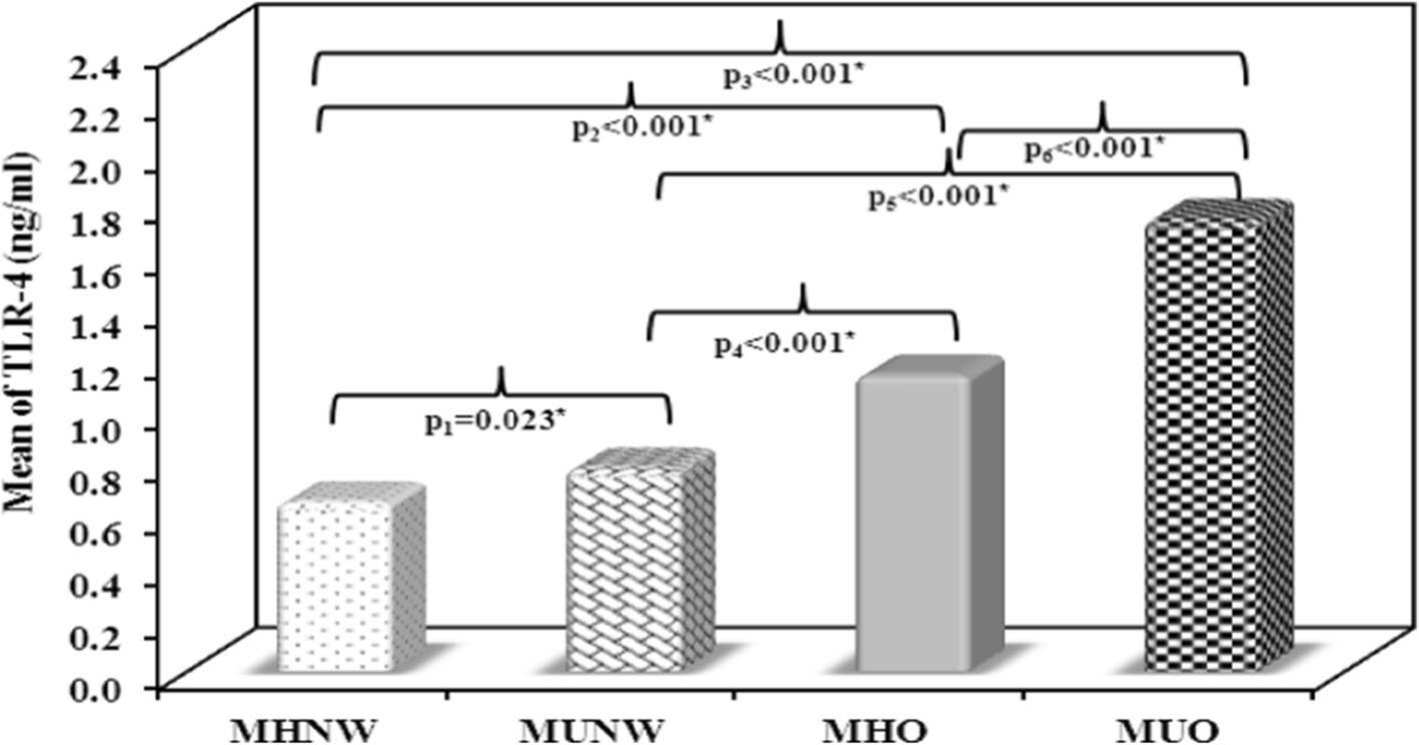
Comparison between the mean values of serum Toll like receptor-4 (TLR-4) (ng/mL) in the studied groups. MHNW Metabolically healthy normal weight. MUNW Metabolically unhealthy normal weight. MHO Metabolically healthy obese. MUO Metabolically unhealthy obese. ANOVA test, post hoc test (Tukey) was used for pairwise comparison. p_1_, significant difference MUNW vs. MHNW. p_2_ significant difference MHO vs. MHNW. p_3_ significant difference MUO vs. MHNW. p_4_ significant difference MHO vs. MUNW. p_5_ significant difference MUO vs. MUNW. p_6_ significant difference MUO vs. MHO. *Statistical significance at p < 0.05

**Fig. 4:**
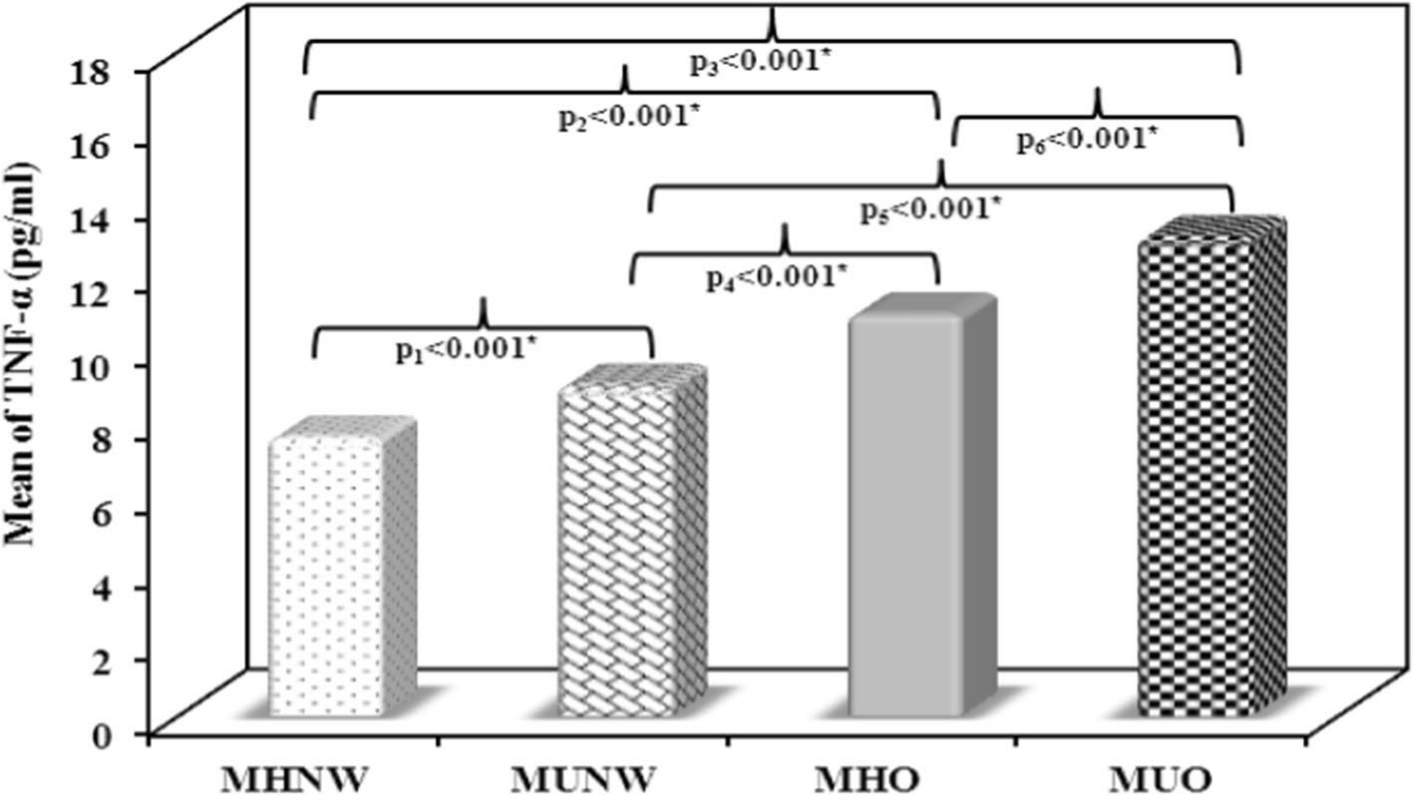
Comparison between the mean values of serum tumor necrosis factor (TNF)-α (pg/mL) in the studied groups. MHNW Metabolically healthy normal weight. MUNW Metabolically unhealthy normal weight. MHO Metabolically healthy obese. MUO Metabolically unhealthy obese. ANOVA test, post hoc test (Tukey) was used for pairwise comparison. p_1_ significant difference MUNW vs. MHNW. p_2_ significant difference MHO vs. MHNW, p_3_ significant difference MUO vs. MHNW. p_4_ significant difference MHO vs. MUNW. p_5_ significant difference MUO vs. MUNW. p_6_ significant difference MUO vs. MHO. *Statistical significance at p < 0.05

The MUO group showed the lowest vitamin D levels as compared to other groups. In both the obese subjects and the normal weight subjects, the mean values of 25(OH)D levels were significantly lower in the metabolically unhealthy subgroups as compared to the metabolically healthy ones (p < 0.001, Fig. 1). On the contrary, the MUO group showed the highest levels in TSP1, TLR-4 and TNF-α as compared to other groups. Mean values of TSP1, TLR-4 and TNF-α levels showed significant increase among the metabolically unhealthy subgroup in comparison to the metabolically healthy subgroup of both the obese and the normal weight subjects (Figs. 2, 3, 4).

### Correlation studies

**Fig. 5:**
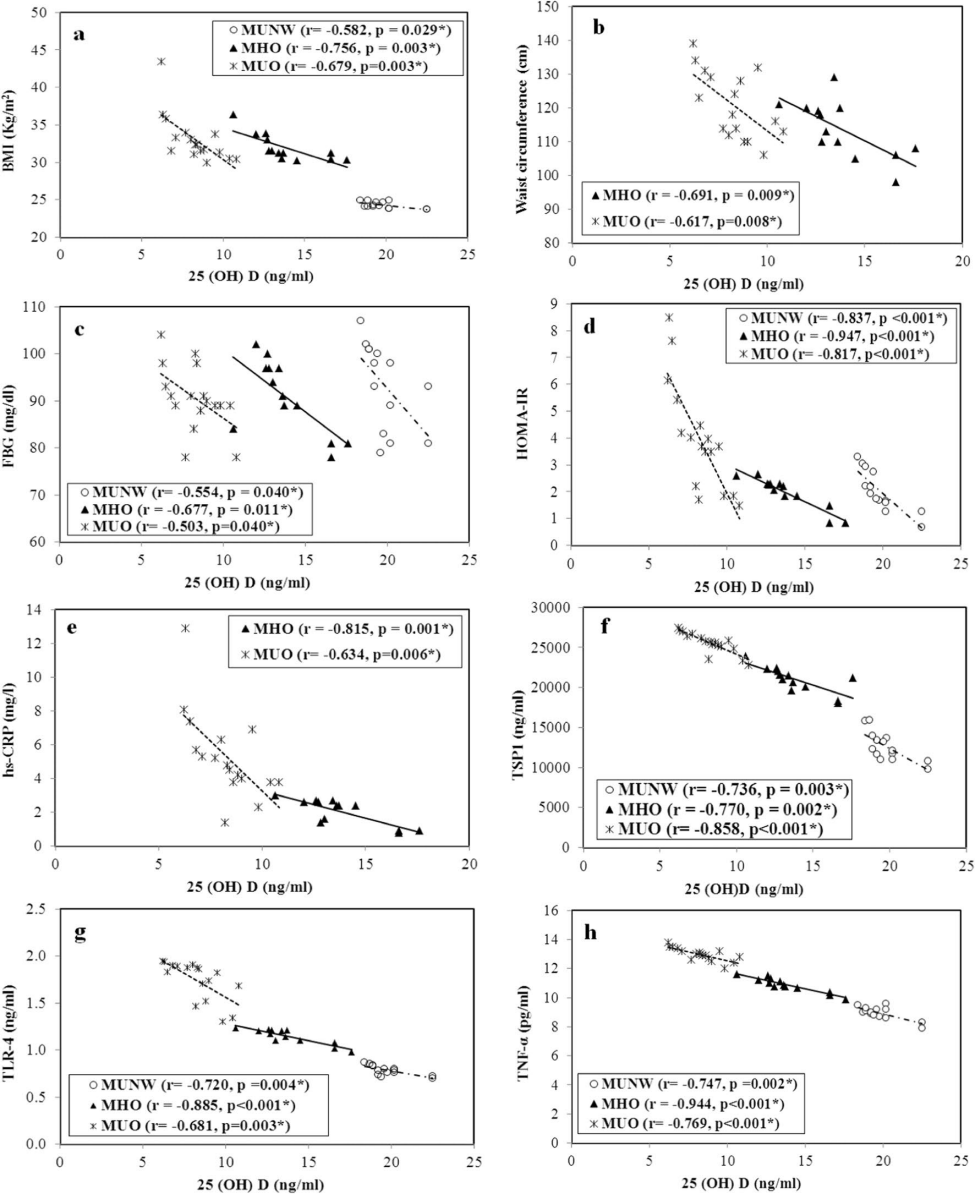
Correlation between serum 25(OH)D (ng/mL) and some studied parameters in the different groups. MUNW Metabolically unhealthy normal weight. MHO Metabolically healthy obese. MUO Metabolically unhealthy obese. **a** BMI Body mass index. **b** Waist circumference. **c** FBG Fasting blood glucose. **d** HOMA-IR Homeostasis model assessment for insulin resistance. **e** hs-CRP High sensitivity C reactive protein. **f** TSP1 Thrombospondin-1. **g** TLR-4 Toll like receptor-4. **h** TNF-α Tumor necrosis factor-α. r Pearson’s coefficient. *Statistical significance at p < 0.05

Correlations of serum 25(OH)D with the other studied parameters are presented in Fig. 5. 25(OH) D levels significantly negatively correlated with each of the following: BMI [r = − 0.582 (p = 0.029), r = − 0.756 (p = 0.003) and r = − 0.679 (p = 0.003)], TSP1 [r = − 0.736 (p = 0.003), r = − 0.770 (p = 0.002) and r = − 0.858 (p < 0.001)], TLR4 [r = − 0.720 (p = 0.004), r = − 0.885 (p < 0.001) and r = − 0.681 (p = 0.003)], TNF-α [r = − 0.747 (p = 0.002), r = − 0.944 (p < 0.001) and r = − 0.769 (p < 0.001)] and HOMA-IR [r = − 0.837 (p < 0.001), r = − 0.947 (p < 0.001) and r = − 0.817 (p < 0.001)] in the MUNW, MHO and MUO group, respectively. 25(OH) D levels significantly negatively correlated with the waist circumference and the hs-CRP levels only in the obese subjects [for the waist circumference r = − 0.691 (p = 0.009) and r = − 0.617 (p = 0.008) and for the hs-CRP levels r = − 0.815 (p = 0.001) and r = − 0.634 (p = 0.006) in the MHO and MUO group, respectively].

**Fig. 6:**
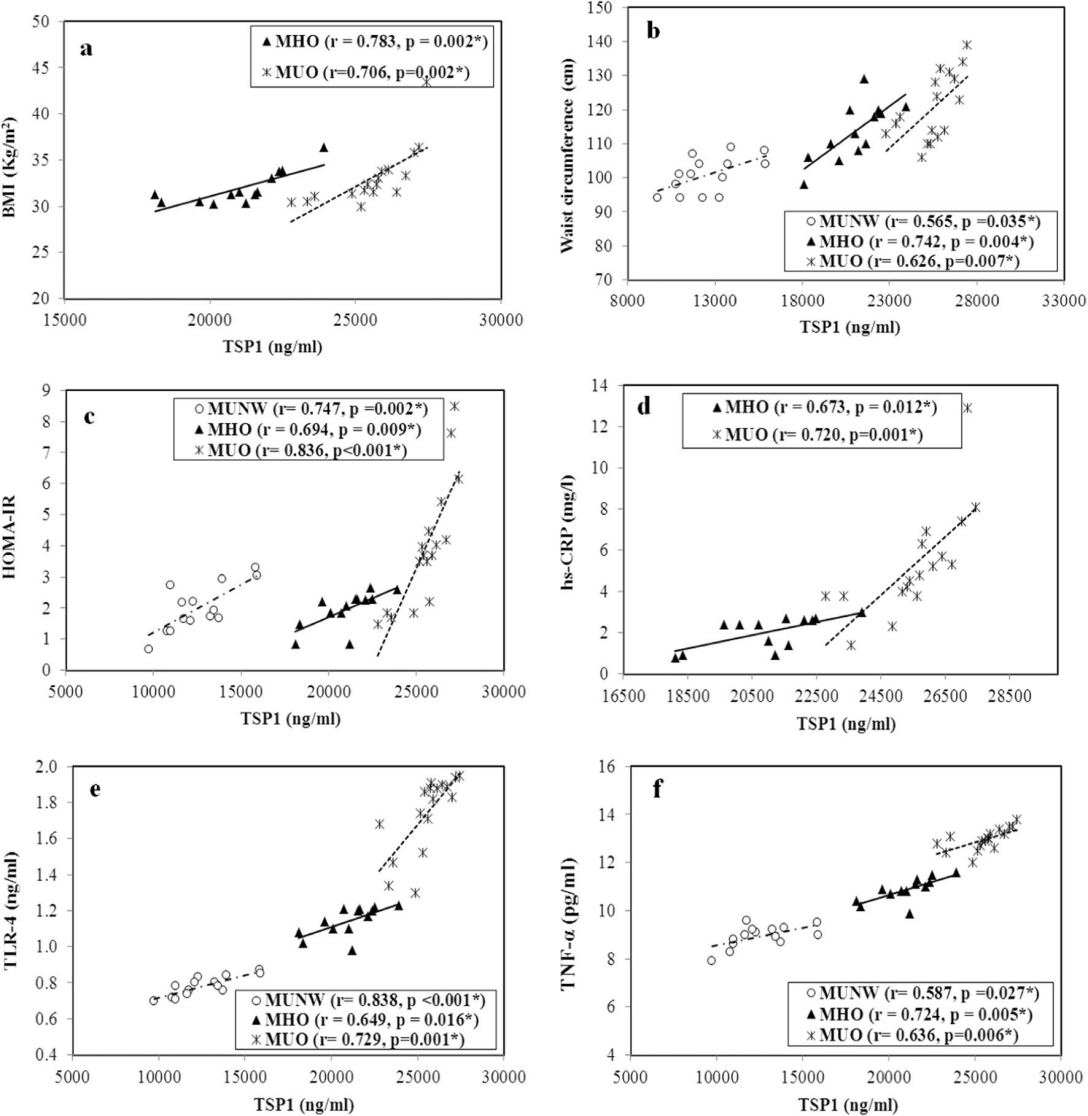
Correlation between serum thrombospondin-1 (TSP1) levels (ng/mL) and some studied parameters in the different groups. MUNW Metabolically unhealthy normal weight. MHO Metabolically healthy obese. MUO Metabolically unhealthy obese. **a** BMI Body mass index. **b** Waist circumference. **c** HOMA-IR Homeostasis model assessment for insulin resistance. **d** hs-CRP High sensitivity C reactive protein. **e** TLR-4 Toll like receptor-4. **f** TNF-α Tumor necrosis factor-α. r Pearson’s coefficient. *Statistical significance at p < 0.05

Additionally, significant positive correlations were found between serum TSP1 and the following parameters: waist circumference [r = 0.565 (p = 0.035), r = 0.742 (p = 0.004) and r = 0.626 (p = 0.007)], TLR-4 [r = 0.838 (p < 0.001), r = 0.649 (p = 0.016) and r = 0.729 (p = 0.001)], TNF-α [r = 0.587 (p = 0.027), r = 0.724 (p = 0.005) and r = 0.636 (p = 0.006)] and HOMA-IR [r = 0.747 (p = 0.002), r = 0.694 (p = 0.009) and r = 0.836 (p < 0.001)] in the MUNW, MHO and MUO group, respectively. Serum levels of TSP1 significantly positively correlated with the BMI and hs-CRP levels only in the obese subjects [for the BMI r = 0.783 (p = 0.002) and r = 0.706 (p = 0.002) in the MHO and MUO, respectively and for the hs-CRP levels r = 0.673 (p = 0.012) and r = 0.720 (p = 0.001) in the MHO and MUO, respectively] (Fig. 6).

## Discussion

The adipokine TSP1 has been shown to have a role in the pathophysiology of the obesity-related inflammation via activation of the TLR4 signaling pathway [10, 13]. A regulatory effect of vitamin D on TSP1 has been suggested [19], however, this effect has not been addressed in different metabolic health status. Given the proposed link between the suboptimal vitamin D status and unfavorable metabolic and inflammatory profiles [16, 28]; the current study assessed the relationship between the TSP1/TLR4 inflammatory pathway and levels of vitamin D in obese and normal weight subjects with different metabolic phenotypes, as a possible anti-inflammatory mechanism by which vitamin D affects metabolic health. Results revealed significantly lower level of serum 25(OH)D and significantly higher levels of serum TSP1, TLR4 and TNF-α in the obese in comparison to the normal weight subjects and in the MUO and the MUNW subjects in comparison to the MHO and the MHNW subjects, respectively, indicating inflammation in obesity as well as in metabolic unhealthy status. TSP1 correlated positively with parameters of unhealthy metabolic profile. Levels of TSP1, TLR4 and TNF-α showed significant negative correlations with serum vitamin D levels in the MUNW, MHO and MUO subjects.

An association between inflammation and impaired metabolic health, both in obese and non-obese individuals, has been previously suggested [3]. In accordance, in the present study, significantly higher serum levels of the inflammatory markers CRP and TNF-α were observed in the metabolically unhealthy subjects (obese or normal weight) compared to their metabolically healthy counterparts. The inflammatory cytokines may affect the metabolic health through several mechanisms. Of these, TNF-α, monocyte chemoattractant protein (MCP)*-*1 and interleukin (IL)-6 were reported to interfere with hepatic insulin signaling via inhibiting the insulin receptor substrate-1 (IRS1) phosphorylation [29, 30]. Moreover, TNF-α enhances adipocyte lipolysis, MCP-1 increases the hepatic triglyceride accumulation and IL-6 suppresses adiponectin expression [31, 32]. Nevertheless, studies comparing MHO and MUO subjects showed conflicting results, where either no difference or higher levels of inflammatory cytokines as IL-6 and TNF-α in the MUO were found [3, 33, 34]. The inconsistent results could be due to differences in gender, age and the used definition of MHO.

In the present study, the observed higher serum levels of TSP1 in obese as well as in metabolically unhealthy subjects is in line with the previously reported over expression of adipose tissue TSP1 in obese and insulin-resistant individuals [10] and in obese mice [11]. Besides, serum TSP1 has been showed to be a useful biological marker of obesity and metabolic syndrome [9].

One possible link between TSP1 and metabolic health could be through its role in regulating the macrophage function [11, 35]. TSP1 deficiency has been demonstrated to be associated with reduced macrophage recruitment and activation and to have a protective effect against obesity-induced inflammation and insulin resistance [11, 12, 35]. On the other hand, TSP1 treatment of the macrophages has been found to promote their expression of TNF-α in a dose and time-dependent manner via activating the TLR4/nuclear factor-kappaB (NF-kB) pathway, partially through interacting with its receptor CD36 and this was suggested as a mechanism by which TSP1 regulates macrophage activity [13]. Furthermore, TSP1-deficiency in mice was recently demonstrated to be associated with reduced expression of the TLR4 and the inflammatory cytokines IL-1β and TNF-α in the liver [36]. In agreement, in the present work, levels of TLR4 and TNF-α were significantly positively correlated with TSP1 levels. The TLR4 could affect the inflammatory reaction by enhancing the inflammatory cytokines such as IL-6 and TNF-α [37].

In the present study, serum TLR4 level was used as a biomarker for the inflammatory status mediated by the membrane-bound TLR4. The circulating form of TLR4 is produced from the conversion of cell-surface TLR4 and believed to have a similar immuno-inflammatory regulatory role [38]. Serum TLR4 has been reported to be a potential biomarker associated with several inflammatory conditions [39–41].

Previous studies have detected higher expression of TLR4 and TNF-α in obese as compared to lean subjects with a remarkably higher expression in obese and overweight individuals with type 2 diabetes [42, 43]. Moreover, TLR4 gene deletion has been showed to protect against insulin resistance in mice fed with a high fat diet, thus pointing at a causal role played by TLR4 in metabolic dysregulations [44].

Therefore, the observed higher levels of TSP1, TLR4 and TNF-α, in the current study, among the obese subjects as well as the metabolically unhealthy ones either obese or normal weight could confirm the role of inflammation in predisposing to disturbed metabolic health and may suggest the role of this inflammatory pathway in regulating adipose tissue macrophage function that could be implicated in the metabolic dysregulations.

As regards the relation of vitamin D with TSP1 levels, an inverse relationship has been found between the levels of serum TSP1 and serum vitamin D in children with sickle cell anemia [21]. Moreover, vitamin D supplementation was reported to decrease TSP1 levels and to downregulate TSP1 mRNA expression [19, 20], suggesting a negative regulatory action exerted by vitamin D on TSP1. In similarity, the present study confirmed this relation between vitamin D and TSP1 levels and indicated the role of vitamin D in regulating TSP1 levels in healthy and unhealthy metabolic status in obese subjects and also in MUNW subjects.

The pathogenic mechanisms responsible for the deficiency of vitamin D in the obese subjects include sequestration in the adipose tissue, volumetric dilution, diminished synthesis of vitamin D in the adipose tissue and limited exposure to sunlight [15, 45]. In addition, fat accumulation in the abdominal area is predictive of vitamin D deficiency. The observed negative correlation between the waist circumference and serum vitamin D levels indicates that fat distribution is more crucial than the total body fat amount and that the presence of abdominal obesity is considered a predictive factor for the worsening of vitamin D deficiency. Szymczak-Pajor et al. [46] have reported that although the adipose tissue represents one of the main stores of vitamin D, vitamin D is deficient in obese subjects. The molecular responses to vitamin D in the adipose tissue are involved in energy metabolism, anti-inflammatory cytokines and adipokines production, antioxidant defense and adipocytes differentiation via regulating genes expression. Thus, vitamin D deficiency disturbs adipogenesis, lipid storage, metabolism and the regulation of adipocytokines secretion and oxidative stress balance [46].

Vitamin D deficiency has been recognized as a risk factor for metabolic disorders [47]. In accordance, in the present work, lower vitamin D levels were detected in the obese compared to the non-obese subjects as well as in the subjects with unhealthy metabolic profile compared to the metabolically healthy subjects, as serum 25(OH)D levels were significantly reduced in the MUNW in comparison to the MHNW subjects and in the MUO in comparison to the MHO subjects. Additionally, 25(OH)D levels showed significant inverse correlations with most of the parameters of unhealthy metabolic profile, hence confirming the role played by vitamin D in maintaining metabolic health. Some studies have reported lower levels of 25(OH)D in the MUO versus MHO subjects, they suggested that vitamin D deficiency could represent a key component in the MUO phenotype [48, 49]. The deficiency of vitamin D in obese subjects was reported to be associated with abnormal metabolic components and abnormal inflammatory biomarkers with a suggested important role for vitamin D receptor (VDR) gene polymorphisms in altering the immune and inflammatory status [28]. On the contrary, other studies found non-significant difference between the levels of 25(OH)D in the MHO and the MUO [50, 51], in spite of the reported inverse associations between the levels of 25(OH)D and triglycerides levels, insulin resistance and blood pressure [50], this could be explained by differences in the degree of obesity or in the criteria used for defining metabolic health.

The association of vitamin D with insulin sensitivity could be attributed to the involvement of vitamin D in insulin secretion and fat metabolism. Both the pancreatic β-cells and the adipocytes express VDR [52]. Also, vitamin D enhances the expression of insulin receptors on target tissues, in turn improves insulin sensitivity [53]. Moreover, vitamin D reduces the activity of the NF-kB signaling pathway [54], thus vitamin D deficiency decreases peripheral insulin sensitivity as a result of an increased systemic inflammation [17]. These could explain the inverse relation of vitamin D with HOMA-IR in the present study.

## Conclusions

In summary, findings of the current study showed significantly lower levels of vitamin D and significantly higher levels of TSP1, TLR4 and TNF-α in obese subjects compared to normal weight subjects and in metabolically unhealthy subjects compared to metabolically healthy subjects, thus confirmed the relation of both reduced vitamin D levels and enhanced TSP1/TLR4 pathway with inflammation and disturbed metabolic health. TSP1 correlated positively with parameters of unhealthy metabolic profile. Besides, vitamin D levels significantly negatively correlated with levels of TSP1, TLR4 and TNF-α, suggesting a negative regulatory effect exerted by vitamin D on this pathway, thus providing a potential mechanism linking hypovitaminosis D with the risk of inflammation-induced metabolic dysfunctions.

Screening for obesity and metabolic health should be routinely done. Vitamin D supplementation may represent a promising intervention for improving the dysfunctional adipose tissue and preventing transition from a metabolically healthy to a metabolically unhealthy status. Further clinical and follow up studies are warranted to establish the benefits of restoring vitamin D levels in obese or normal weight subjects with different metabolic phenotypes.

## Data Availability

The datasets generated during and/or analyzed during the current study are available from the corresponding author on reasonable request.
